# Netrin-1 Stimulates Developing GnRH Neurons to Extend Neurites to the Median Eminence in a Calcium- Dependent Manner

**DOI:** 10.1371/journal.pone.0046999

**Published:** 2012-10-09

**Authors:** Victoria F. Low, Zeno Fiorini, Lorryn Fisher, Christine L. Jasoni

**Affiliations:** Centre for Neuroendocrinology, Department of Anatomy, University of Otago, School of Medical Sciences, Dunedin, New Zealand; Institut National de la Recherche Agronomique-CNRS UMR6175, France

## Abstract

Hypothalamic gonadotropin-releasing hormone (GnRH) neurons are required for fertility in all mammalian species studied to date. In rodents, GnRH neuron cell bodies reside in the rostral hypothalamus, and most extend a single long neuronal process in the caudal direction to terminate at the median eminence (ME), the site of hormone secretion. The molecular cues that GnRH neurites use to grow and navigate to the ME during development, however, remain poorly described. Reverse transcription-PCR (RT-PCR) identified mRNAs encoding *Netrin-1*, and its receptor, *DCC,* in the fetal preoptic area (POA) and mediobasal hypothalamus (MBH), respectively, from gestational day 12.5 (GD12.5), a time when the first GnRH neurites extend toward the MBH. Moreover, a subpopulation of GnRH neurons from GD14.5 through GD18.5 express the Netrin-1 receptor, DCC, suggesting a role for Netrin-1/DCC signaling in GnRH neurite growth and/or guidance. In support of this notion, when GD15.5 POA explants, containing GnRH neurons actively extending neurites, were grown in three-dimensional collagen gels and challenged with exogenous Netrin-1 (100 ng/ml or 400 ng/ml) GnRH neurite growth was stimulated. In addition, Netrin-1 provided from a fixed source was able to stimulate outgrowth, although it did not appear to chemoattract GnRH neurites. Finally, the effects of Netrin-1 on the outgrowth of GnRH neurites could be inhibited by blocking either L-type voltage-gated calcium channels (VGCCs) with nifedipine (10 µM), or ryanodine receptors with ryanodine (10 µM). This is consistent with the role of Ca2+ from extra- and intracellular sources in Netrin-1/DCC-dependent growth cone motility in other neurons. These results indicate that Netrin-1 directly stimulates the growth of a subpopulation of GnRH neurites that express DCC, provide further understanding of the mechanisms by which GnRH nerve terminals arrive at their site of hormone secretion, and identify an additional neuronal population whose neurites utilize Netrin-1/DCC signaling for their development.

## Introduction

Hypothalamic gonadotropin-releasing hormone (GnRH)-secreting neurons are required for fertility in all mammals studied to date [1], [2]. GnRH neurons in the mouse are generated from progenitors in the olfactory placode beginning around gestational day (GD) 9.5 [3], [4] and GnRH gene expression commences shortly thereafter at GD 10.75 for the first-born cells [4]–[7]. Following differentiation, GnRH neurons migrate from their site of generation into the brain, then, once inside the brain, they take a ventro-caudal path to their final site of residence in the rostral hypothalamus [8]–[13]. Much attention has been paid to understanding these early phases of GnRH neuron development, and a clear picture of the underlying molecular and cellular mechanisms is emerging (for reviews see [12], [14]–[17]).

In order for GnRH neurons to play their normal hypophysiotrophic role, their processes must extend from the somata, in the rostral hypothalamus, to the median eminence (ME), in the mediobasal hypothalamus (MBH). By contrast with the wealth of data on GnRH neuron differentiation and migration, far less is known about the mechanisms underlying the targeting of GnRH neuronal processes to the ME. Several diffusible factors, including fibroblast growth factor 2 (FGF2) [18]–[20], brain-derived neurotrophic factor (BDNF) [21], the Prader-Willi gene candidate *necdin* [22], and the puberty-driving Kisspeptin peptide [23], have been implicated in GnRH process growth *in vitro* and/or *in vivo*.

The establishment of correct neuronal circuitry in the brain is achieved through the integrated functions of several families of highly conserved guidance cues. One such cue is Netrin-1, which is, along with its receptors, including DCC (deleted in colon cancer), is expressed throughout the developing brain and spinal cord, and provides key navigational information for both commissural as well as longitudinal neuronal process growth [24], [25]. Global deletion of either Netrin-1 or DCC results in a massive reduction in GnRH innervation of the ME [26]. Analysis of the expression pattern of Netrin-1 showed that it was expressed along the rostral-caudal hypothalamic midline, including the ME, and thus is in the correct place to play a role in GnRH migration and/or axon guidance [26]. Subsequent studies that examined GnRH neuron development in more detail demonstrated that global deletion of either Netrin-1 or DCC resulted indirectly in failed GnRH migration because these mutants had a disrupted olfactory axon trajectory, which GnRH neurons normally use as a scaffold on which to migrate into the brain [26]–[28]. Consequently, much of the loss of ME innervation by GnRH neurites seen in Netrin-1 or DCC mutants could be explained by a reduced number of GnRH neuron cell bodies in the brain. Thus, an intriguing speculation arising from these investigations [26], and a remaining unanswered question, concerns whether Netrin-1 normally acts as a diffusible cue that stimulates growth and/or provides navigational information to GnRH neurites as they travel to the ME.

The study reported here was designed to address this question, by asking whether Netrin-1 provides growth and/or guidance cues to GnRH neuroendocrine processes en route to the ME. To do this we initially defined the spatial and temporal expression patterns of DCC and Netrin-1 in and around, respectively, GnRH neurons in the brain during the time of their process extension, and then evaluated whether Netrin-1 could modulate GnRH process growth and/or guidance. We found that: 1) the Netrin-1 receptor DCC was expressed in a subpopulation of GnRH neurons during the time of their process extension; 2) Netrin-1 was expressed in the POA and ME across development; 3) Netrin-1 stimulated the growth of, but did not chemoattract, GnRH neurites from fetal POA; 4) The effects of Netrin-1 were attenuated in the presence of either the L-type calcium channel blocker nifedipine, or the intracellular Ca^2+^ store-release blocker ryanodine. Together these data indicate that Netrin-1 provides growth signals to a subpopulation of GnRH neurites through DCC-dependent calcium influx from intra- and extracellular pools.

## Methods

### mRNA Extraction and Reverse Transcription-PCR

All animal procedures performed in this study were approved by the University of Otago Animal Ethics Committee. Timed pregnant female transgenic GnRH-GFP [29] mice were killed by cervical dislocation on GD 12.5, 14.5, 16.5 or 18.5, where the day of vaginal plug was counted as GD 0.5. Embryos were dissected from the uterus, decapitated, and MBH or POA tissue was manually dissected from the brains in ice-cold phosphate-buffered saline (PBS; 3 mM Na_2_HPO_4_, 1 mM NaH_2_PO_4_, 150 mM NaCl, pH 7.4). The POA was identified as the region of the anterior hypothalamus ventral to the anterior commissure extending rostrally from the caudal limit of the medial septum to the optic chiasm. MBH was dissected as the ventralmost tissue lying approximately 250 µm to either side of the midline and approximately 250 µm to either side of the pituitary stalk. The latter, owing to its highly vascular nature, is clearly visible even at these early ages.

Tissue from each age was pooled, and total RNA was extracted using the Trizol reagent (Invitrogen, Australia) as per manufacturer’s protocol. RNA yield was determined on a Nanodrop spectrophotometer (Thermo Scientific). Extracted RNA was then treated with DNase I (DNA-*free*™ kit, Ambion) as per manufacturer’s protocol, to remove residual genomic DNA and reduce the potential for false-positive results in subsequent PCR. DNase-treated total RNA (5 µg) was then used in a reverse transcription (RT) reaction with Invitrogen Superscript III First-strand synthesis supermix (Invitrogen, Australia) using the manufacturer’s recommended protocol. Negative control samples contained 5 µg total RNA, all the same reaction components and were processed identically, but lacked reverse transcriptase. GD 12.5 cervical spinal cord was processed in parallel to serve as a positive control, since Netrin-1 and DCC expression have been described previously in this area in vertebrates [30], [31].

One µl of RT was then used in a 25 µl PCR containing the following components: 1× buffer (supplied with *Taq* polymerase), 1.5 mM MgCl_2_, 0.2 mM each of dATP, dCTP, dGTP, dTTP (Invitrogen, Australia), 25 nmole each primer (Invitrogen), and 20 U Platinum *Taq* polymerase (Invitrogen, Australia). Reactions were cycled in using standard parameters with a 60°C annealing temperature. The freeware program Primer3 (http://frodo.wi.mit.edu/) was used primer selection. For *Netrin-1* RT-PCR, primer sequences were based on the NCBI reference sequence NM_008744 and were: 5′-TGGTGACCAGAGTTTGTGGA-3′ (forward); 5′-TTCTTGCACTTGCCCTTCTT-3′ (reverse). For *DCC* RT-PCR, primer sequences were based on the NCBI reference sequence NM_007831 and were: 5′-GAATGAGGCATGTGCTCAGA-3′ (forward); 5′-AGAAGGCTCCAAAGGAGAGG-3′ (reverse). PCR products (219 bp Netrin-1, 174 bp DCC) were visualized on a standard 1× TAE (40 mM Tris-base, 0.1% acetic acid, 1 mM EDTA, pH 7.4) 1% agarose gel, alongside a 1 kb plus ladder (Invitrogen, Australia) and digitally photographed using a SynGene (SynOptics Ltd., Cambridge, UK) gel documentation system. For publication, gel photos from several independent PCRs were cut from the original photos and pasted together to form a composite image using Adobe Photoshop v12.0.1 x64 (Adobe Systems Inc., USA). Decorations were added with Adobe Illustrator v15.0.1 (Adobe Systems Inc., USA).

**Figure 1:**
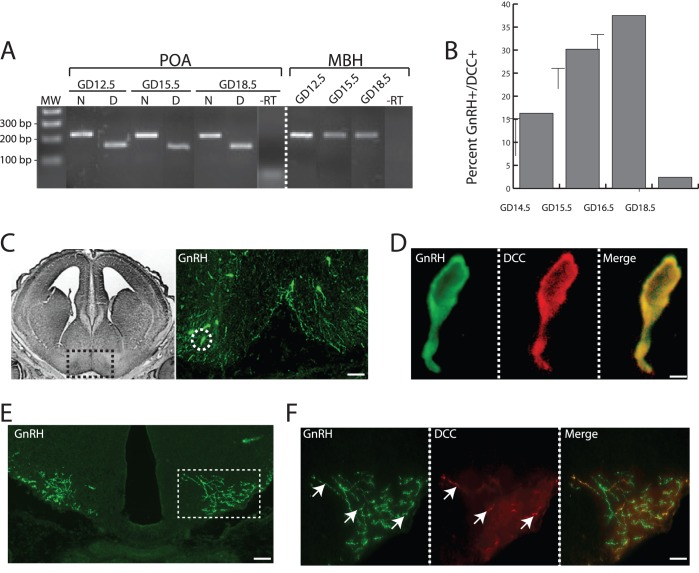
Expression of Netrin-1 and its receptor DCC in developing GnRH neurons and target areas. (A) Composite image showing agarose gel electrophoresis of PCR products. PCR from the preoptic area (POA), across all ages examined (GD 12.5, 15.5, 18.5), showed a band of 219 bp arising from Netrin-1-specific primers (lanes marked with N), and a band of 174 bp arising from DCC-specific primers (lanes marked with D). MW, molecular weight marker. PCR from the mediobasal hypothalamus (MBH) was performed with primers for Netrin-1 only, and again a band of 219 bp is clearly visible. –RT, negative control lanes showing that there are no PCR products from reactions in which the reverse transcription reaction contained no reverse transcriptase, and verifying the specificity of the reactions and the absence of spurious products. (B) Bar graph showing the mean percentage of GnRH-immunoreactive neurons that were also immunoreactive for DCC across the three gestational ages when GnRH neurites are growing to the median eminence. Error bars indicate SEM. (C) Left, black and white image modified from The Chemoarchitectonic Atlas of the Developing Mouse Brain [52] section of GD 16 mouse brain, and depicting the anatomical level from which the immunostained section (right) was taken. Black box indicates region that is shown in the accompanying immunofluorescence photo. Right, representative image of GD 16.5 coronal brain section showing immunostained GnRH (green) neurons (arrows). Dotted white circle indicates one GnRH neuron that is shown dual-labeled with DCC and at higher magnification in (D). Scale bar, 100 µm. (D) High magnification of a single GnRH neuron showing dual-labeling for GnRH peptide (green), DCC (red), two channels merged (yellow). Scale bar, 10 µm. (E) Representative image of GD 16.5 coronal brain section at the level of the ME, and showing immunostained GnRH nerve fibers (green). Dotted box demarcates stained fibers, and indicates the area that is shown in higher magnification in (F). Scale bar, 100 µm. (F) High magnification of GnRH nerve fibers at the ME showing dual-labeling for GnRH peptide (green), DCC (red), and the two channels merged. Arrows indicate GnRH fibers that are co-expressing DCC protein. Scale bar, 10 µm.

### Dual-label Immunohistochemistry

Fetal brains were collected as described, fixed by immersion in 2% paraformaldehyde in phosphate buffered saline (PBS; 0.1 M sodium phosphate, 0.9% NaCl) for 2 hours at room temperature, cryopreserved by incubation in a 50∶50 mixture of 30% sucrose:OCT (Optimal Cutting Temperature solution; Leica, Australia) overnight at 4°C, frozen in sucrose:OCT mixture, cryosectioned at 20 µm in the coronal plane, and collected onto gelatin-coated slides. Tissue sections were subjected to dual-label immunocytochemistry with mouse anti-DCC (AF5, Abcam, USA) diluted 1∶1,000 in incubation solution (0.5% Triton-X100 (Sigma-Aldrich, Australia), 1% bovine serum albumin (Sigma-Aldrich, Australia)), and rabbit anti-GFP (Molecular Probes, A11122, Invitrogen, Australia) diluted 1∶10,000 in incubation solution, plus an additional 2% normal goat serum (NGS; Vector labs, Burlingame, CA, USA) and 2% normal donkey serum (Sigma-Aldrich, Australia). In previous experiments [23], we validated that GFP expression was a faithful indicator of GnRH neurons and their processes in explants taken from GnRH-GFP mice [29].

Primary antibodies were incubated with explants for 24–48 h at 4°C, and then washed 3 times in PBS for 1 hr each at room temperature. Secondary antibodies, goat anti-rabbit-Alexa-488 (Invitrogen, Australia) and donkey anti-mouse-Alexa-594 (Invitrogen, Australia) were diluted 1∶1,000 in incubation solution and incubated for 90 min at room temperature, protected from light. Explants were then washed 3 times in PBS for 1 h each at room temperature, and imaged on a Zeiss LSM-510 inverted confocal within 48 h of completing the immunocytochemistry.

**Figure 2:**
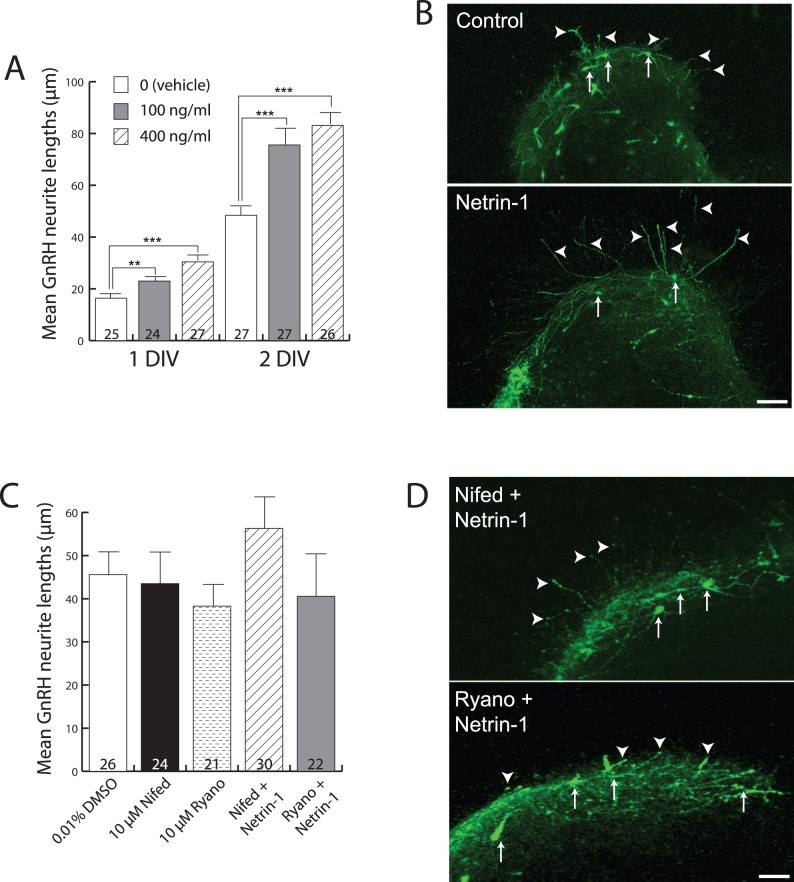
Netrin-1 stimulates the growth of GnRH neurites in a calcium-dependent manner. (A) Bar graph showing mean GnRH neurite lengths at 1 and 2 DIV across the three different treatments (indicated in inset legend). Numbers at the base of each bar indicate the number of explants used, and are from fetal brains derived from 5 independent pregnancies (average of 8 fetuses/pregnancy). Error bars indicate SEM. **, *P*<0.01 and ***, *P*<0.001 with one-way ANOVA and Student-Newman-Keuls multiple comparison post-hoc test. (B) Immunofluorescence images for detection of GFP peptide as a surrogate marker for GnRH neurons (arrows) of explants grown for 2 DIV. Note the clear difference in the length of GnRH neurites (arrowheads) outside the explant borders in Netrin-1-treated (lower panel) by comparison with untreated (upper panel). Scale bar, 40 µm. (C) Bar graph showing mean GnRH neurite lengths (y-axis) at 2 DIV under the three treatment conditions indicated on the x-axis. Numbers at the base of each bar indicate the number of explants used, and are from fetal brains derived from 4 independent pregnancies. Abbreviations: Nifed = nifedipine, Ryano = ryanodine. Error bars indicate SEM. (D) Immunofluorescence images showing GFP-expressing GnRH neurons grown in explants in the presence of Netrin-1 plus either the L-type VGCC blocker Nifedipine (Nifed + Netrin-1, upper panel) or ryanodine (Ryano + Netrin-1, lower panel). Arrows indicate GnRH neuron cell bodies, and arrowheads indicate GnRH nerve fibers extending beyond the borders of the explants. Scale bar, 100 µm.

### Neurite Outgrowth and Guidance Assessment

Fetal brains were collected as above, and were placed in ice-cold Leibowitz (L15, Invitrogen) containing 4.5 g/L D-glucose. The POA was dissected free (as described above), and divided into four approximately 200 µm^3^ pieces. In pilot experiments, GnRH-GFP hypothalami were dissected under epifluorescence microscopy to reveal the position of embryonic POA GnRH neurons, which showed green fluorescence, and thereby precisely define the area to be dissected. Explants were rinsed three times in fresh sterile L15 with glucose, before being embedded in a 3-dimensional collagen gel.

Collagen gels were prepared as described previously [23], [32], [33]. Briefly, 1 mg/ml (0.1%) collagen (type I from rat tail, BD Biosciences) solution in 1× medium [consisting of 1× DMEM/F12 (Invitrogen, Australia), 10 mM HEPES (Sigma-Aldrich, Australia), 25 mM NaHCO_3_ (Sigma-Aldrich, Australia), 1× B27 (Invitrogen, Australia), 10% fetal bovine serum (Bio International Ltd., Hamilton, New Zealand), 1% D-glucose (Sigma-Aldrich, Australia), 1% Penn/Strep (Invitrogen, Australia), pH 7.4], and containing either 100 ng/ml or 400 ng/ml carrier-free recombinant mouse Netrin-1 (R & D Systems, Minneapolis, MN, USA), or vehicle (sterile PBS) was prepared on ice and aliquoted (250 µl) to cover the bottom of each well of a 24-well plate (Corning, Australia). The collagen was then allowed to solidify in a humidified 37°C/5% CO_2_ incubator for at least 20 min prior to addition of the explants. Netrin-1 was reconstituted in vehicle (sterile PBS) to a final stock concentration of 100 µg/ml. For all experiments Netrin-1 was used within 2 months of reconstitution. To determine suitable concentrations of Netrin-1 we consulted the literature to ensure that the doses of Netrin-1 were in keeping with current practice. Deiner et al., 1997, reported a dose-response study with recombinant Netrin-1 that showed maximal effectiveness on neurite growth at 100 ng/ml. Similar doses were reported elsewhere [34], [35]. For the L-type VGCC inhibition experiments, 100 mM nifedipine (Sigma-Aldrich, Australia), reconstituted in DMSO (Sigma-Aldrich, Australia), was added to the collagen gel mix to a final concentration of 10 µM. For the ryanodine treatment experiments, ryanodine (Tocris, UK) was reconstituted in 100% ethanol to a stock concentration of 10 mM, and diluted into the collagen gel mix to a final concentration of 10 µM. Explants were placed in the center of the collagen gel base, overlaid with 25 µl of the appropriate collagen solution (described above), and then cultured in a humidified 37°C/5% CO_2_ incubator for 24–48 hrs.

**Figure 3:**
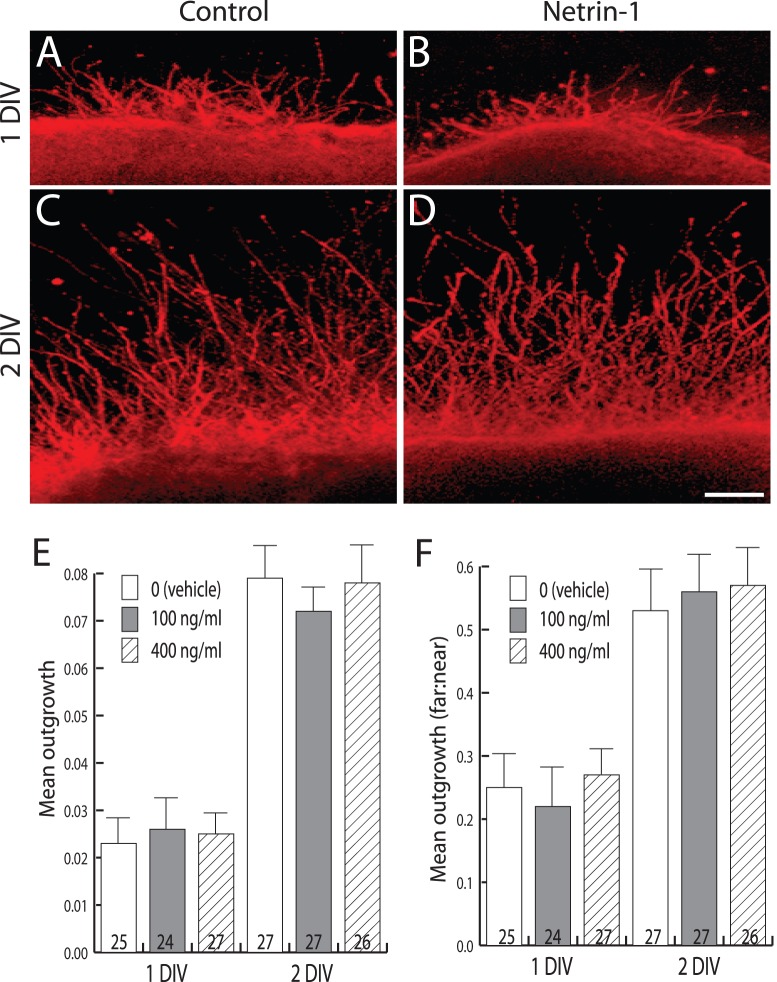
Netrin-1 does not affect the outgrowth of TuJ1^+^ neurites in POA explants. (A-D) Representative images of explants cultured as indicated for 1 or 2 DIV in the presence of 100 ng/ml Netrin-1 (Netrin-1) or control. Scale bar, 70 µm. (E) Bar graph showing the mean outgrowth of TuJ1+ neurites (y-axis) across indicated culture conditions (x-axis) at 1 or 2 DIV. Numbers at the base of each bar indicate the number of explants used, and are from fetal brains derived from 5 independent pregnancies. Error bars indicate SEM. (F) Bar graph showing the mean far: near ratio (y-axis) for neurite lengths across culture conditions (x-axis) as indicated by inset legend. Decorations as in E. Error bars indicate SEM.

For experiments in which Netrin-1 was provided from a fixed source, collagen gels were prepared as above, but without the addition of Netrin-1 or vehicle. Agar blocks (1% agar (Sigma-Aldrich, Australia) in PBS, approximately 500 µm^3^) were soaked in 10 µg/ml Netrin-1 in medium for 1–2 h on ice. For this agar block method, it has been reported that factor concentration drops off significantly outside the agar block [33]. Thus, it is usual for a high concentration (up to 1000 times greater) of chemoattractive factor within the agar block to be used in order to achieve an effective concentration at the neurites extending from the explant. Control blocks were soaked in parallel in medium alone. Agar blocks were placed in the center of the collagen gel base, three explants were arranged around the agar block at no more than 300 µm away (average distance between agar block Netrin-1 source and explant was 270 µm), and collagen was overlaid. Explants prepared in this way were cultured for 36–40 h in a humidified 37°C/5% CO_2_ incubator. For the L-type VGCC inhibition experiments, 10 µM nifedipine was added to the collagen gel mix. For the ryanodine receptor inhibition experiments, ryanodine was added to the collagen gel mix to a final concentration of 10 µM.

### Explant Immunocytochemistry

Fixed explants were subjected to dual-label immunocytochemistry with antibodies to beta-III tubulin (TuJ1, monoclonal, R & D Systems, Minneapolis, MN, USA) diluted 1∶1,000 in incubation solution (0.5% Triton-X100 (Sigma-Aldrich, Australia), 1% bovine serum albumin (Sigma-Aldrich, Australia)), or mouse anti-DCC (AF5, Abcam, USA) diluted 1∶1,000 in incubation solution (0.5% Triton-X100 (Sigma-Aldrich, Australia), 1% bovine serum albumin (Sigma-Aldrich, Australia)), along with rabbit anti-GFP (Molecular Probes, A11122, Invitrogen, Australia) antibody diluted 1∶10,000 in incubation solution (0.5% Triton-X100 (Sigma-Aldrich, Australia), 1% bovine serum albumin (Sigma-Aldrich, Australia)), plus an additional 2% normal goat serum (NGS; Vector labs, Burlingame, CA, USA). In previous experiments [23], we validated that GFP expression was a faithful indicator of GnRH neurons and their processes in explants taken from GnRH-GFP mice [29].

**Figure 4:**
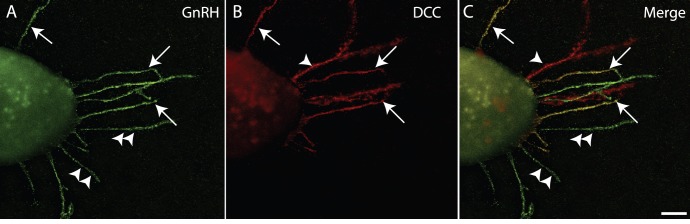
DCC is expressed by a subpopulation of GnRH neurites extending from POA explants *in vitro*. Example images from dual-label immunocytochemistry to detect GnRH (green, left panel), DCC (red, middle panel), and two colors merged (right panel) performed on explants that had been grown for 2 DIV. Note that three different classes of neurites were visualized: GnRH^+^/DCC^+^ (arrows), GnRH^+^/DCC^-^ (double arrowheads), and GnRH^−/^DCC^+^ (arrowheads). Scale bar, 20 µm.

Primary antibodies were incubated with explants for 24–48 h at 4°C, and then washed 3 times in PBS for 1 hr each at room temperature. Secondary antibodies, goat anti-rabbit-Alexa-488 (Invitrogen, Australia) or and donkey anti-mouse-Alexa-594 (Invitrogen, Australia) were diluted 1∶1,000 in incubation solution, and incubated for 90 min at room temperature, protected from light. Explants were then washed 3 times in PBS for 1 h each at room temperature, and imaged on a Zeiss LSM-710 inverted confocal within 48 h of completing the immunocytochemistry.

### Explant Imaging

Each explant was imaged in 4–6 sub-regions at 10× magnification, with a fixed Z plane thickness of 50 µm. The regions comprising the explant were then merged (using ImageJ64, v1.46 a) to form a montage of the explant. All montaged images were then coded so that the experimenter was blind to their identity until after the measurements (below) had been performed. For publication, the levels of the individual sub-regions were adjusted individually using Adobe Photoshop 12.0.1 x64 (Adobe Systems, Inc., USA) to give a resulting image devoid (or nearly so) of obvious subdivisions. Montage images thus prepared were imported into Adobe Illustrator v15.0.1 (Adobe Systems Inc., USA) for the addition of decorations.

**Figure 5:**
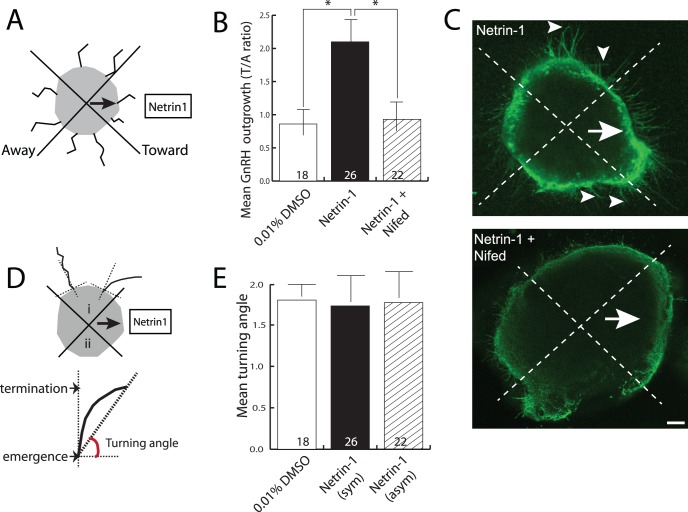
Netrin-1 does not chemoattract GnRH neurites. (A) Schematic diagram showing the fixed source with explant set-up and definition of toward and away sides. (B) Bar graph showing the toward:away (T/A) ratio of the lengths of GnRH neurites (y-axis) as a function of treatment (x-axis). Numbers at the base of each bar indicate the number of explants used, and are from fetal brains derived from 3 independent pregnancies. Error bars indicate SEM. *, *P*<0.05 with Mann-Whitney test of significance. (C) Example of explants cultured for 2 DIV in the presence of a fixed source of Netrin-1, either with (lower panel) and without (upper, control) nifedipine, and immunostained to reveal GnRH neurons and their processes. The arrow indicates the toward side (ie., the direction of the Netrin-1 source), and arrowheads indicate representative GnRH^+^ neurites. Scale bar, 40 µm. (D) Schematic drawing of turning angle analysis. Upper indicates two possible orientations in which neurites may grow as they exit the explant, either growing relatively straight (left) or turning toward the Netrin-1 source (right). i and ii indicate the two quadrants from which neurites were counted. Lower indicates how turning angle was measured, where emergence refers to the point at which the neurite exits the explant (set to 0), and termination indicates the position of the terminal swelling that demarcates the end of the neurite. (E) Bar graph showing the mean turning angle (y-axis) of GnRH neurites extending from the explant across the conditions indicated (x-axis). Error bars represent SEM. Numbers at the base of each bar indicate the number of explants used, and are from fetal brains derived from 3 independent pregnancies. Abbreviations, Netrin-1 (sym) indicates explants that were exposed to a symmetrical Netrin-1, Netrin-1 (asym) indicates explants that were exposed to asymmetrical Netrin-1 from a fixed source.

### GnRH Neurite Measurements and Statistical Analysis

The lengths of individual GnRH neurites were measured from their emergence at the explant border to their tips using the ImageJ64 plugin, NeuronJ, which uses a “live-wire” segmentation paradigm to trace elongated structures, such as neurites, in 2 dimensional images [36]. To ensure that the terminus of the neurite was being labeled, only neurites with a clear morphological termination – either a growth cone (majority) or bouton-like terminal swelling – were counted and measured. Neurite lengths were averaged across multiple explants within each experimental condition to get a mean ± SEM neurite length for each condition, and these were then compared statistically across conditions using one-way ANOVA with Student-Newman-Keuls multiple comparison post-hoc test (InStat, GraphPad Software, Inc). Although the mean number of visible GnRH neurons did not vary significantly from explant to explant, their distribution, or position, relative to the explant border did differ among explants. To ensure that this did not affect the analysis or interpretation, explants were only included if they had ≥10 GnRH neurons within 100 µm (approximately 2–3 cell diameters) of the explant border. Since DCC-expressing GnRH neurons seemed evenly distributed, there was no additional normalization for this subpopulation.

Neurites from explants that were exposed to an asymmetric gradient of Netrin-1 or vehicle (PBS) were imaged and measured in the same manner. Mean ± SEM neurite length and number were calculated for two regions, one toward the source (T) and the other, of equal area, away from the source (A). These measurements were then used to compute the guidance ratio *R* = *(T – A)/(T + A),* as described previously [23], [33]. Because *R* is a ratio that is normalized for total outgrowth *(T + A)*, it should be insensitive to variability in total outgrowth among explants. Guidance ratios for explants exposed to Netrin-1 versus vehicle were then compared statistically using a two-tailed non-parametric pairwise Mann-Whitney test (InStat, GraphPad Software, Inc).

In addition to guidance ratio, the turning angle was also computed as the difference between the angle of emergence (set to 0) and the angle at termination relative to the position of the ager block (see for example [37], [38]). At least 25 randomly selected neurites were counted from intermediate quadrants for each explant.

### TuJ1^+^ Neurite Measurements and Statistical Analysis

For each explant, the TuJ1^+^ neurite outgrowth was quantitated in two ways. First, using the methods described by Rosoff et al. (2004) [33], ImageJ was used to outline the border of each explant, and the image outside of the explant was thresholded and converted to a binary image. The total number of non-zero pixels outside the explant was divided by the number of pixels in the explant. Outgrowth was then averaged for all explants in a given condition to arrive at a mean ± SEM for each condition, and these were then compared statistically across conditions using one-way ANOVA with Student-Newman-Keuls multiple comparison post-hoc test (InStat, GraphPad Software, Inc.). A second calculation was also used for assessing TuJ1^+^ neurites in order to capture better the length element of their growth. For this calculation, the TuJ1^+^ pixel intensity was measured in a 50 µm x 50 µm area at 5 randomly chosen locations within the first 100 µm from the perimeter of the explant. These measurements were then averaged to arrive at a mean (± SEM) pixel intensity, and therefore TuJ1^+^ neurite density, for the area (near). This was repeated for 5 locations in the area between 100 µm and 200 µm from the explant perimeter (far), and then the ratio of far:near was calculated to arrive at a number that represents the average relative TuJ1^+^ extension. Importantly, both approaches rely on the assumption that fluorescence intensity is a proxy for immunoreactive neurite number, and thus gives information about the relative growth of neurites from explants, not individual neurite lengths, which are very difficult to measure individually due to the high density of fibers extending outside the explant.

## Results

### Expression of Netrin-1 and DCC in the Developing Hypothalamus

RT-PCR and immunohistochemistry were used to ascertain the expression patterns of *Netrin-1* and *DCC* mRNAs and DCC protein, respectively, across the developmental window when GnRH neurons extend neurites to the ME. Both *Netrin-1* and *DCC* mRNAs were present in the developing preoptic area (POA) and *Netrin-1* was present in the mediobasal hypothalamus (MBH) from GD12.5 through GD18.5 (Fig. 1A). Given that GnRH neurons arrive in the POA, cease migration, and extend processes toward the ME during this developmental time, these observations suggest a role for Netrin-1/DCC signaling in migration and/or neurite extension to the ME.

To determine whether GnRH neurons specifically express the Netrin-1 receptor, DCC, and thus have the potential to respond to Netrin-1, we used dual-label immunofluorescence to detect GnRH peptide and DCC across the time of GnRH nerve fiber extension. DCC was expressed in a subpopulation of GnRH neurons at GD14.5 (16.3% ±1.2%), GD15.5 (30.2% ±2.3%), GD16.5 (37.5% ±5%), and GD18.5 (2.4% ±0.6%) (Fig. 1B), but was absent from adult GnRH neurons (not shown). DCC peptide could be visualized most clearly within the cell body (Fig. 1C, D), but was also clearly evident in a subpopulation of GnRH^+^ neurites in or near the ME (Fig. 1E, F). There was no significant difference in distribution of DCC-expressing GnRH neurons across the rostro-caudal GnRH neuron continuum (not shown). Finally, there was no evidence that migrating GnRH neurons (those present in the nasal area or within the brain in regions rostral to the MS) showed DCC expression at any age examined (not shown). This temporal pattern of DCC expression by GnRH neurons is strikingly similar to the time course of GnRH neurite extension to the ME [20], [39], and supports a role for Netrin-1/DCC signaling in this process.

### Netrin-1 Stimulates Hypothalamic GnRH Neurite Outgrowth in vitro in a Calcium-dependent Manner

The temporal pattern of Netrin-1 expression relative to GnRH nerve fiber growth, and the finding that DCC is expressed by 30–40% of GnRH neurons when they are actively extending neuronal processes *in vivo*, led us to speculate that Netrin-1 may have the ability to affect the growth of neurites from GnRH neurons. To test this hypothesis a 3-dimensional (3D) collagen gel assay [33], which has been used previously by our group to examine GnRH neurite growth [23], was employed. GD15.5 POA explants were used, because this age represents the developmental time when the largest proportion of GnRH neurons are actively extending their neurites [20].

GnRH neurite outgrowth was increased significantly in the presence of Netrin-1 (100 ng/ml or 400 ng/ml), after 1 DIV and 2 DIV (Fig. 2A, B). At 1 DIV, 100 ng/ml Netrin-1 increased GnRH process length from 16.4 µm ±1.4 (n = 25 explants, vehicle) to 23.0 µm ±1.4 (n = 24 explants, *P*<0.01), and 400 ng/ml Netrin-1 increased GnRH process length to 30.4±1.8 (n = 27 explants, *P*<0.001). Similarly, at 2 DIV, 100 ng/ml Netrin-1 increased GnRH process length from 48.4 µm ±2.4 (n = 27 explants, vehicle) to 75.6 µm ±3.7 (n = 27 explants, *P*<0.001), and 400 ng/ml Netrin-1 increased GnRH process length to 83.1±2.9 (n = 26 explants, *P*<0.001). There was no significant difference in GnRH process length between 100 ng/ml and 400 ng/ml at any DIV. These observations, together with the expression of DCC in GnRH neurons, suggest that GnRH neurites are directly responsive to growth stimulatory signals provided by Netrin-1.

In many neuronal cell types, stimulatory Netrin-1/DCC signaling is associated with the influx of calcium ions (Ca^2+^) from both extracellular and intracellular sources, which initiates further downstream events, such as cytoskeletal changes, associated with both growth cone advance and turning [40], [41]. To evaluate whether Ca^2+^ influx was similarly involved in the growth-stimulating effects of Netrin-1 on GnRH neurites, the above experiments at 2 DIV were repeated in the presence of blockers for L-type VGCCs and ryanodine receptors. In the presence of the L-type VGCC blocker nifedipine (10 µM) alone GnRH neurite lengths (43.5 µm ±4.4) were slightly, but not significantly, reduced compared with vehicle (0.01% DMSO)-treated control lengths (45.6 µm ±4.7) (Fig. 2C). Similarly, in the presence of the store release blocker ryanodine (10 µM) alone, GnRH neurite lengths (38.3 µm ±5.1) were again slightly, but not significantly, reduced compared with vehicle-treated control lengths, indicating that ryanodine did not have a marked affect on basal outgrowth of GnRH neurites. Together these control results indicate that blocking Ca^2+^ influx from either extracellular or intracellular pools did not have a marked affect on basal outgrowth of GnRH neurites. Importantly, the control level of GnRH neurite outgrowth in the presence of 0.01% DMSO was not significantly different from untreated explants (compare Fig. 2A and 2C), indicating that this concentration of DMSO was not itself affecting GnRH neurite growth.

When POA explants were grown in the presence of Netrin-1 (400 ng/ml) plus nifedipine (10 µM) GnRH neurite lengths remained at control levels (57.2 µm ±7.1), although there was a slight trend for increased lengths. When POA explants were grown in the presence of Netrin-1 plus ryanodine (10 µM), GnRH neurite lengths similarly remained at control levels (40.0±10.1). Together, these data indicate that the stimulatory effects of Netrin-1 on GnRH neurite growth are dependent on Ca^2+^ influx from both the extracellular space, through L-type VGCCs, and intracellular stores, via ryanodine receptors.

We next were curious to determine whether other cells in the explants may also be responsive to Netrin-1, which could indicate that some GnRH neurites may respond indirectly to Netrin-1 via its effects on other cells present in the explant. To address this we first determined the ability of Netrin-1 to stimulate outgrowth from TuJ1^+^ (a pan neuronal marker) neurons. By contrast with GnRH neurites, the average outgrowth of GD15.5 POA TuJ1^+^ neurites at either 1 or 2 DIV was unchanged by the presence of Netrin-1 (Fig. 3). At 1 DIV, 100 ng/ml Netrin-1 produced a mean outgrowth ratio of 0.026±0.005, and 400 ng/ml produced a mean outgrowth ratio of 0.025±0.008, and neither was significantly different from explants without Netrin-1 whose mean outgrowth ratio was 0.023±0.006 (Fig. 3A, B, E). Similarly, at 2 DIV the presence of Netrin-1 at either 100 ng/ml (outgrowth ratio of 0.072±0.005) or 400 ng/ml (outgrowth ratio of 0.078±0.009) produced no significant differences in growth compared to untreated explants with an outgrowth ratio of 0.079±0.007 (Fig. 3C, D, E). Because total pixel intensity outside the explant may be due to increased neurite length, branching, or both, an alternate measurement (far:near ratio) was used to capture better the length element of the neurite growth. Theoretically, early in the culture period, when most neurites are expected to be quite short and not extend much into the “far” region, the far:near ratio would be quite small. However as neurites grow longer, and an increased number occupy the far region, this ratio would grow larger. Using this calculation, no changes were observed in TuJ1^+^ neurite length in the presence of Netrin-1. At 1 DIV, 100 ng/ml Netrin-1 produced a mean far:near ratio of 0.22±0.07, and 400 ng/ml Netrin-1 produced a mean far:near ratio of 0.27±0.05, and neither was significantly different from explants without Netrin-1 whose mean far:near ratio was 0.25±0.07 (Fig. 3 A, B, F). Similarly, at 2 DIV the presence of Netrin-1 at either 100 ng/ml (far:near ratio of 0.56±0.07) or 400 ng/ml (far:near ratio of 0.57±0.07) produced no significant differences in growth compared to untreated explants (far:near ratio of 0.53±0.08) (Fig. 3 C, D, F). Together these observations of TuJ1^+^ outgrowth indicate that Netrin-1 is not affecting the growth of a majority of neurites in the culture, and are consistent with a direct effect of Netrin-1 on GnRH neurite growth.

It is likely that the TuJ1+ neurite population may itself be composed of a mix of Netrin-1-responsive and non-responsive neurites, which, if measured together, may give an overall impression of non-responsiveness even though there may be non-GnRH Netrin-1-responsive neurites present. To ascertain whether there were non-GnRH neurites expressing DCC, and to get an idea of their relative proportions, we assessed the expression of DCC in the explants. Fig. 4 shows a 2 DIV explant with several GnRH^+^ neurites extending from it (Fig. 4A, C). Dual labeling for DCC revealed that some (∼30%) GnRH neurites expressed DCC, and others did not (Fig. 4B, C). Importantly, there did not appear to be many non-GnRH neurites expressing DCC in these POA explants, which supports the interpretation that Netrin-1 affects only a subpopulation of GnRH neurites, and is not affecting the growth of a majority of neurites in the culture.

### Netrin-1 is not Chemoattractive to GnRH Neurites

Because Netrin-1 acting via DCC is chemoattractive to neurites of many developing neuronal types [30], [42]–[44], we sought to determine whether Netrin-1 might be similarly chemoattractive to developing GnRH neurites. To test this, we provided a fixed asymmetric source of Netrin-1 (in the form of an agar block soaked in Netrin-1) to POA explants, and then assayed for whether GnRH neurites preferentially extended toward the Netrin-1 source. Diffusion of Netrin-1 from the fixed source establishes a concentration gradient of Netrin-1 similar to long-range chemoattraction *in vivo,* and is a routinely used assay for chemoattraction/repulsion activity from a variety of neurite growth regulators [45]–[47] (see Fig. 5A, D for schematic design). Although GnRH neurons were evenly distributed over the entire explant, GnRH neurites preferentially extended from the side of the explant facing the Netrin-1 source. The number of GnRH neurites emanating from the side of the explant facing toward the Netrin-1-soaked agar block (T/A ratio = 2.1±0.7, n = 26) was significantly increased relative to vehicle-only controls (T/A ratio = 0.86±0.4, n = 18, *P*<0.05), or explants treated with Netrin-1 plus 10 µM nifedipine (T/A ratio = 0.9±0.5, n = 22, *P*<0.05) (Fig. 5A, B, C).

Although the number of neurites emerging from the explant on the side with the Netrin-1 source was significantly greater compared with controls, such growth may be due to an increase in rate of growth or sprouting on the Netrin-1 side, rather than to turning of neurites toward the source as would be expected for chemoattraction. Thus, an independent assessment of chemoattraction that quantitates whether the GnRH neurites turn toward the Netrin-1 source (the so called turning angle) was also employed. This measure is usually employed for isolated neurons growing in a gradient of guidance cue, where the growth trajectory of the neurite in the absence of factor is compared with the growth trajectory in the presence of factor, typically in a time-lapse scenario. To measure this in the POA explants the angle at which the neurite exited the explant was set to zero, and its final angle relative to starting angle was calculated (depicted in Fig. 5B). Explants exposed to a fixed asymmetric source of Netrin-1 (turning angle 1.77±0.43), showed no significant reorientation of their neurites when compared to neurites grown in either vehicle (1.80±0.24) or Netrin-1 that was symmetrically surrounding the entire explant (1.73±0.37). This indicates that Netrin-1 promotes the growth of GnRH neurites, but does not have a powerful chemoattractive influence, and is consistent with inhibition by nifedipine (Fig. 5B, C) or ryanodine (not shown).

## Discussion

We have shown that a subpopulation of developing GnRH neurons express the Netrin-1 receptor, DCC, in both their cell bodies and neuroendocrine processes *in vivo*. Using *in vitro* axon growth and guidance assays, we further demonstrated that Netrin-1 stimulated the growth of GnRH neurites in a calcium-dependent manner, but did not appear to provide a chemoattractive influence on this neuronal population. Together this work addresses the question originally posed by Deiner and Sretavan [26] by demonstrating that Netrin-1/DCC signaling plays a role in GnRH neuron development through its ability to stimulate the growth of GnRH neurites as they travel to the ME.

Global deletions of either DCC or Netrin-1 show massive reductions in the number of GnRH neurons in the hypothalamus [26]–[28]. This phenotype appears to be due to a disruption of the olfactory axon trajectory on which GnRH neurons migrate, leading to a much reduced number of GnRH neurons making it into the brain, and subsequently in a reduced number of GnRH nerve fibers in the ME. Thus, it remained an open question whether Netrin-1/DCC signaling had a direct effect on GnRH neurite growth. The data presented here extend previous reports by quantifying DCC expression within the developing GnRH population across developmental time. This supports the conclusion that Netrin-1/DCC signaling directly within GnRH neurons occurs during the period of process extension. Moreover, this is consistent with the prior suggestion that loss of Netrin-1/DCC signaling in neurite growth and navigation may contribute to the absence of GnRH nerve fibers at the ME seen in DCC or Netrin-1 deletion mutants [26]–[28].

It is curious that only a subpopulation of GnRH neurons at any given developmental stage express DCC. There are two factors that might account for this, and they may not be mutually exclusive. First, because GnRH neurons go through the processes of differentiation, migration and neurite extension each across multi-day windows of development, only a subpopulation of GnRH neurons would be extending neurites on any given day. Given the time course of GnRH neuron migration, and assuming that migration is immediately followed by neurite extension as it is in other neurons [48], the peak of GnRH neurite extension in the mouse should be the window from GD15.5 to GD17.5. Prior and subsequent to this window the proportion of GnRH neurons extending neurites is expected to be lower, and thus the proportion of GnRH neurons expressing neurite growth regulators would also be lower. The percentage of GnRH neurons expressing DCC across development that we have observed here is consistent with this interpretation. Secondly, heterogeneity within the adult GnRH neuron population is well-documented [1]. Given this, it may not be surprising to find heterogeneity among developing GnRH neurons as well. Moreover, it has been suggested that heterogeneity in GnRH neuron developmental history may be a causal explanation for their adult heterogeneity [7], [16], although a clear link has yet to be established. Thus, even if our observations were restricted only to GnRH neurons extending neurites, it is not unreasonable to suspect that this population may itself be heterogeneous in its gene expression patterns, including that of DCC. There is likely to be functional significance to this heterogeneity in DCC expression. One possibility is that the DCC^+^ GnRH neurons could act as pioneers that extend to the ME in a Netrin-1/DCC-dependent manner. Such pioneers might be imagined to demarcate a pathway that the DCC-negative GnRH neurites follow to the ME. Alternatively, they may have no influence on DCC-negative GnRH neurites, which would navigate to the ME via some other mechanism. This latter alternative might be imagined to provide a level of robustness to allow fertility to be retained should Netrin-1/DCC signaling be perturbed within post-migratory GnRH neurons. In support of this, examination of DCC and GnRH expression in the neurites extending from POA explants showed little evidence of apparent consistent physical interaction between DCC^+^ and DCC^-^ GnRH neurons. The ability to remove DCC specifically from GnRH neurons post-migration would add further support to this mechanism.

Although asymmetric Netrin-1 promoted asymmetric GnRH neurite growth from the POA explants, the analysis of turning angle failed to show a clear ability of Netrin-1 to reorient, and thus chemoattract or repulse, GnRH neurites. Because the true starting angle of growth cannot be determined from within the explant, this measurement is likely to be an underestimate of the ability of Netrin-1 to cause reorientation or turning of GnRH neurites. Further studies that examined the ability of Netrin-1 to influence the direction of isolated GnRH neurites (e.g. by using the pipette assay [49], [50]) could potentially clarify this issue.

Previously, Deiner and Sretavan (1999) [26] raise the point that a number of developmental abnormalities all seem to overlap anatomically at the optic chiasm, perhaps suggesting that developmental defects in this region may have secondary effects on developmental processes, such as GnRH migration, positioning, and subsequent neurite extension, that may rely on the chiasm for patterning or other instructional information. Although the data presented here have used isolated GnRH neurons along with some POA tissue for in vitro investigation, there remains the issue of what happens *in vivo*. There is no denying that the optic chiasm is a powerful patterning center. The idea that numerous cell types may use the chiasm as a source of patterning cues seems highly likely, and is indeed a plausible explanation for the array of anatomical abnormalities (including those of GnRH migration and neurite extension) that are seen in global deletions of genes, including DCC [26], involved in chiasm formation/patterning. Deiner and Sretavan (1999) [26] aimed to determine the role of Netrin-1/DCC signaling in ventral hypothalamic development, to address whether this region may be analogous molecularly to the ventral midline of more caudal brain areas. They observed that axon projections of retinal ganglion cells and GnRH neurons were much reduced, and that several hypothalamic cell types failed to migrate to their proper locations. The ability to delete molecules of interest, such as DCC, specifically from GnRH neurons would be an elegant approach to removing the possibility of indirect effects on GnRH neuron development consequent to a previous developmental defect. Another approach would be to affect the formation of the chiasm specifically through mutation of genes that are not involved in the development of GnRH neurons. We have attempted to address this by examining Pax2 mutants, in which the chiasm does not form properly [51], and found that the number and placement of GnRH neurons as well as their innervation of the ME were unaffected, despite the complete lack of an optic chiasm (unpublished observations).

The data presented here suggest that Netrin-1/DCC signaling within a subpopulation of post-migratory GnRH neurons stimulates their growth in a manner that is critically dependent on Ca^2+^ influx from both intracellular and extracellular sources. This work identifies Netrin-1/DCC signaling as another key molecular player in ensuring that GnRH neurites grow to ME, the site of hormone secretion. How the various growth and navigation factors that have been identified work together normally, and whether (and how) such cooperativity ensures that fertility is not compromised by a single developmental mistake, remain as intriguing further questions to resolve.
